# Recycling potential of carbon fibres in the construction industry: From a technical and ecological perspective

**DOI:** 10.1177/0734242X241237197

**Published:** 2024-04-17

**Authors:** Berfin Bayram, Vanessa Overhage, Marco Löwen, Katharina Terörde, Karoline Raulf, Kathrin Greiff, Thomas Gries

**Affiliations:** 1 Department of Anthropogenic Material Cycles, RWTH Aachen University, Aachen, Germany; 2 RWTH Aachen University, Institut für Textiltechnik, Aachen, Germany; 3 RWTH Aachen University, Aachen, Germany

**Keywords:** Carbon fibre, recycling, concrete, life cycle assessment, circular economy

## Abstract

Even though carbon fibres (CFs) have been increasingly used, their end-of-life (EOL) handling presents a challenge. To address this issue, we evaluated the use of recycled CFs (rCFs), produced through pyrolysis, as rovings to be used in textile reinforced concrete structures. Mechanical processing (hammer mill) with varying machine settings was then used to assess EOL handling, considering the separation potential of rCFs and the length of separated rCFs. The results showed that rCF rovings can be separated from concrete with an average of 87 wt.-%, whereas the highest rCF length and separation yield were observed in different machine settings. In addition, a techno-environmental assessment on the mechanical process was performed to compare different machine settings. The machine settings with the highest yield of rCF rovings also had the highest fine fraction that cannot be further separated. Furthermore, life cycle assessment (LCA) was conducted covering three life cycles of CFs and an additional LCA for comparing rCF with virgin CF. LCA results revealed that CF reinforced plastic and concrete productions are the two main contributors to environmental impacts. The comparative LCA between virgin CF and rCF also showed that using rCF is environmentally advantageous, as virgin CF production causes 230% more global warming potential compared to rCF. Future studies assessing different allocation approaches, quantifying the quality of rCF, and its inclusion in LCA are relevant.

## Graphical abstract

**Graphical Abstract:**
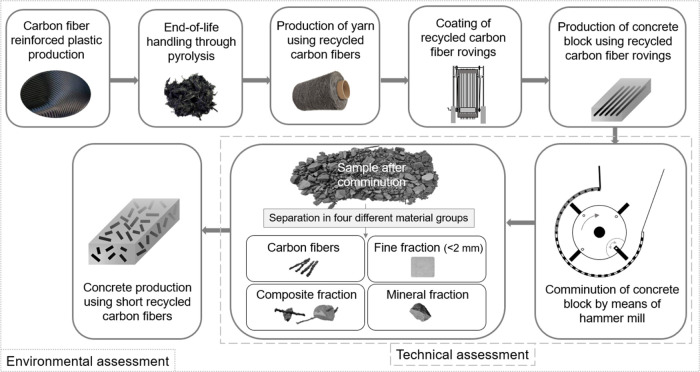
This is a visual representation of the abstract.

## Introduction

The market share of carbon fibres (CFs) has been increasing globally (Bledzki et al., 2021; Di He et al., 2020). One of the application areas of CFs is the construction industry, where CF-reinforced concrete (CFRC) enables a great amount of resource savings due to its light weight, high strength and durability (Backes et al., 2022a; Böhm et al., 2018; Reichenbach et al., 2021; Stoiber et al., 2021). These advantages are highly pertinent to the concept of sustainable raw material use, since reducing resource usage and increasing product lifespan play a central role. However, despite these benefits, CFs also present some drawbacks, such as their high cost, energy-intensive production processes and challenges associated with end-of-life (EOL) handling, as the circularity is highly dependent on EOL handling (Zhang et al., 2020). Especially the EOL handling of CF is highly problematic for achieving the goals of circular economy (CE) as the fibre length of recycled CF (rCF) is shortened, which considerably limits the further fields of application. Pyrolysis, a thermal recycling process, and solvolysis, a chemical recycling process, are the two mostly employed EOL treatment methods for CFs (Bledzki et al., 2021). However, when it comes to recycling of CFRC, an ideal solution has yet to be developed, and at present mainly downcycling is taking place (Backes et al., 2022a). Downcycling is a widely used term, but it is hardly defined (Helbig et al., 2022). Therefore, we would like to refer to Helbig et al. (2022), in which downcycling is defined as the phenomenon of quality reduction of materials reprocessed from waste relative to their original quality.

With the growing market share of CFs, CFs have also come to the attention of researchers. For instance, Wang et al. (2023) and Karuppannan Gopalraj and Kärki (2020) conducted reviews of EOL handling options for CFs covering both currently available recycling options such as mechanical recycling, thermal recycling (e.g. pyrolysis) and chemical recycling (e.g. solvolysis) as well as recently developed approaches such as degradable resins and the use of supercritical fluids. Naqvi et al. (2018) provided overview of the studies on the recycling of carbon and glass fibre reinforced composites by pyrolysis with a detailed assessment of the technical challenges and reuse options in high-performance composites. The authors also stated that pyrolysis is one of the most suitable recycling methods for the EOL treatment of CF in terms of economic and technical feasibility (Oliveux et al., 2015; Overcash et al., 2018; Pickering, 2006; Pimenta and Pinho, 2011; Wong et al., 2017). Another novel approach was introduced by López et al. (2013), where the authors assessed the recyclability of CFs through a combined process of thermolysis and gasification and examined optimum temperature and gasification time.

For the quantification for environmental impacts, some studies performed life cycle assessment (LCA) (Karuppannan Gopalraj et al., 2021), which is a relevant and widely used approach, on the EOL handling of CFs. Witik et al. (2013) compared pyrolysis, incineration and landfilling of CF-reinforced composite waste as different EOL handling options and concluded that the environmental potential of recycling is highly dependent on the virgin material replaced, which in turn depends on technical limitations and the market condition. Again, focusing on CF-reinforced composite waste, Wei and Hadigheh (2022) compared various EOL handling options both from economic and environmental point of view and concluded that solvolysis, catalytic pyrolysis and pyrolysis combined with oxidation are the most favourable options. Similarly, Vo Dong et al. (2018) assessed four EOL handling options, grinding, pyrolysis, microwave treatment and supercritical water, for CF reinforced plastic (CFRP) waste and concluded that there is a negative correlation between cost and environmental impacts. As there are many different methods for the EOL processing of CF composites, Supplemental Table S1 contains a glossary list of the terms used in the cited literature for the EOL processing of CF composites.

Considering the use of CF in construction industry, Backes et al. (2022a) conducted a comparative LCA on steel and CFRC focusing on EOL treatment options and concluded that EOL handling of steel reinforced concrete has lower impact compared to CFRC. The authors mentioned that the increased environmental impacts through pyrolysis for the EOL handling of CFRC is mainly due to the high energy requirement, which may be overcome by using renewable energy sources as much as possible (Backes et al., 2022a). Focusing on EOL handling of CFRC, Backes et al. (2022b) compared mechanical and thermal recycling options and stated that mechanical processing causes less impacts compared to pyrolysis; however, it is worth to mention that no avoided impacts were included in the study. In addition, the authors noted that crediting of rCF was not performed due to the lack of accurate energy values for the assessed processes and a quantitative proportion of rCFs (Backes et al., 2022b). There are only a limited number of studies focusing on the use of CFs in concrete structures, and to our knowledge there are no studies to date that assessed the separation potential of rCFs from concrete. Thus, the present study focuses on the use of rCFs as rovings in concrete and then mechanical processing, using hammer mill, of the rCF concrete was performed to assess the separation potential of rCF from the concrete structure. Following this, the separated short rCFs were used in concrete block production. From technical perspective, the focus lies on the EOL handling of rCF concrete, where different machines settings for the hammer mill were tested to observe the effect of the machine settings, based on rotational speed and grate size, on the separation potential of rCFs from concrete and the length of the separated rCFs. In addition, an LCA covering three life cycles of CFs is carried out: (1) CFRP production and EOL handling through pyrolysis, (2) the use of rCFs in concrete as rovings and their EOL handling by mechanical processing and (3) the use of short rCFs separated from the second life cycle and their use in concrete. Finally, a simplified LCA on mechanical processing was performed to compare different machine settings through a techno-environmental assessment. Through this study the following research questions are aimed to be answered:

*Technical assessment*:
*RQ1*: How high is the potential of separating rCFs from concrete by means of mechanical processing using a hammer mill?
*RQ2*: What is the effect of different hammer mill machine settings, based on different combinations of rotation speed and grate size, on the separation potential of CFs from concrete?
*Environmental assessment*:
*RQ3*: What are the environmental impacts of each life cycle and what are the main contributors within each life cycle?
*RQ4*: What is the environmental savings potential of using rCF instead of virgin CFs in concrete?
*Techno-environmental assessment*:
*RQ5*: What is the optimal machine setting in terms of environmental impact during the comminution process, from a gate-to-gate perspective?

## Materials and methods

### Production of rCF textile concrete samples

There are several ways in which rCFs could be used in construction industry, more specifically in concrete structures. Firstly, rCF could be used isotropically in concrete as short fibre reinforcement comparable to virgin short fibres. Virgin short fibres are already being used to improve mechanical properties in concrete. On the one hand, short fibres made of polyvinyl alcohol, polypropylene or E-glass, among others, can be added to the concrete to minimise cracking (Friese et al., 2022). Furthermore, short fibres made of PP, PVA, AR-glass, E-glass and steel additionally improve the mechanical properties such as tensile strength or flexural strength (Friese et al., 2022). Due to the production process, short fibres are added to the concrete without impregnation. This complicates separability and recyclability. Further application options for the rCF to be used in construction industry is currently being investigated in the FaBeR research project (INaB, 2023). Another way to utilise the recycled short fibres is to process them into a yarn. The advantage here is that the impregnated rovings produced using rCF yarn can be aligned and positioned in concrete depending on the application.

Within this article, an experimental study focusing on the utilisation of rCF yarn in the construction industry was carried out. For this, the rCF yarns produced within the scope of the CarboYarn project (Lechthaler et al., 2019) were used. The impregnation of rCF rovings was carried out at the Institut fuer Textiltechnik with a labcoater, using epoxy as an impregnating agent. The rovings were conveyed through the pressed-on rollers for the absorption of the impregnating agent. After this process, the impregnated rCF rovings were cured for 2 days in room temperature before concrete pouring, which was performed following DIN EN 12390-2 (2019). The impregnated rCFs were placed with a distance of 2 cm, and in each concrete sample, five pieces of impregnated rCF rovings were used. In total, 18 concrete samples, with a length of 350 mm, a width of 100 mm and a height of 15 mm, were produced. For the technical part, we did not use textile structure but rather impregnated rCF rovings due to the limited material availability. However, the textile structure with rCFs is planned to be further investigated in a follow-up study.

The test samples for the third life cycle were produced in a similar way. However, instead of long rovings, separated rCF rovings after the comminution process were used as short rCFs in concrete (Section ‘Mechanical processing of rCF textile concrete’). The short rCFs were not isotropically embedded in the concrete, but on one level in the tensile zone and for the main part oriented in the same direction to achieve the greatest possible reinforcement influence.

### Mechanical processing of rCF textile concrete

Mechanical processing started with a comminution process aiming for disintegration of rovings from concrete structure. There are various comminution machines that can be used, such as hammer mill, jaw crusher and impact mill. In this study, an experimental set-up was selected considering the results of Kimm et al. (2018), where authors investigated three different crushing methods and concluded that hammer mill has the best degree of purity, with the lowest composite proportion. Thus, within this study, we decided to use a hammer mill (see Supplemental Figure S1) that was manufactured at Department of Anthropogenic Material Cycles (ANTS) at RWTH Aachen University. The hammer mill used has a drive power of 30 kW, enabling the comminution of hard and soft materials.

In total, six different machine settings, based on different combination of rotational speed and grate size, were tested to find the best option that enables separation of rCFs from concrete. The rotational speed and grate size ranges were decided through a set of preliminary experiments and are shown in Table 1. The machine settings were selected based on trial experiments conducted prior to the actual experiment with the aim of achieving a higher separation yield with a longer length of separated rCFs. Considering the aim, the grate size less than 40 mm was not included in the study as it causes increased amount of fine fraction (less than 2 mm) and reduced rCF length. For each machine setting, the comminution process was performed three times to increase the reliability of the data. Thus, a total of eighteen concrete block samples, three for each machine setting, were analysed.

**Table 1.** Different machine settings of hammer mill used for the experiment.

Machine setting	Rotational speed (min^−1^)	Grate size (mm)
S1	1500	No grate
S2	1500	60
S3	1500	40
S4	2000	No grate
S5	2000	60
S6	2000	40

After the comminution process, the output was sorted into four different fractions. First, the fine fraction smaller than 2 mm was screened out. Then, within the remaining material, rCFs, mineral and composite fractions were manually sorted and weighed. In addition, the length of the separated rCFs was measured by visual assessment using a ruler.

Mechanical processing was assessed considering three different parameters, namely percent mass of each fraction in the output (wt.-%), yield (%) and separated rCF length (cm).

Percent masses of each fraction in the output is calculated based on equations (1) and (2).

(1) $$M_{T}=M_{rCF}+M_{\mathrm{composite}}+M_{\mathrm{mineral}}+M_{\mathrm{fine}}$$

(2) $$\mathrm{Percent}\;\mathrm{mass}\;{\mathrm{fraction}}_{i}\;\left(\mathrm{wt}.-\%\right)=\frac{M_{i}}{M_{T}}x\;100$$

$M_{T}$ : Total mass of the output after comminution covering all four fractions.

$M_{rCF}$ : Mass of the separated rCF after comminution.

$M_{\mathrm{composite}}$ : Mass of the composite fraction.

$M_{\mathrm{mineral}}$ : Mass of the mineral fraction that is separated from rCFs after comminution.

$M_{\mathrm{fine}}$ : Mass of the fine fraction after comminution with a particle size of less than 2 mm.

$M_{i}$ : Mass of a specific fraction (rCF, mineral, composite or fine) in the output after comminution.

Yield (%) is used to assess separation potential of the targeted material, in this study rCFs, which is calculated as shown in equation (3).

(3) $$\mathrm{Yield}\;\left(\%\right)=\frac{M_{i,\;output}}{M_{i.\;input}}\times 100$$

$M_{i,\;\mathrm{output}}$ : Mass of the targeted material in the output after comminution.

$M_{i,\;\mathrm{input}}$ : Mass of the targeted material in the input before comminution.

As the last parameter, the length of the rCF that were separated through the comminution process were measured by visual assessment, using a ruler. In total, five different length ranges (. . .<1 cm, 1 cm<. . .<2 cm, 2 cm<. . .<3 cm, 3 cm<. . .<4 cm, and 4 cm<. . .) were set for assessing the results. It is worth noting that the aim was to achieve the longest rCF length (cm) separated by comminution process with the highest yield (%) possible.

### Life cycle assessment

LCA was conducted in accordance with ISO 14040 (2006) and ISO 14044 (2006), following four main steps of LCA. The first step, goal and scope definition, is the core of the LCA, as most decisions for the LCA are made in this step, for example setting the goal of the study, the system boundaries, and the functional unit (FU). In the second step, life cycle inventory (LCI), the data required for modelling is collected, and documentation of assumptions and data sources. In the third step, the life cycle impact assessment (LCIA) methods are applied for the selected impact categories and the LCI data are processed in LCIA. The use of LCA software with integrated LCIA methods accelerates this step. The final step is the interpretation of the results, and it is suggested to include sensitivity and uncertainty analyses. The LCA framework is an iterative process that can be initiated over again if necessary. Each LCA step specific to this study is explained in detail in the following subsections, covering all relevant information.

#### Goal and scope

The aim of this study is to quantify the environmental impacts of three life cycles of CFs and perform a hotspot analysis to assess the main contributors within each life cycle. Furthermore, an assessment on the potential savings of using rCF instead of virgin CFs was conducted. Lastly, a simplified LCA for the techno-environmental assessment for the comparison of different machine settings was performed.

**Figure 1.**
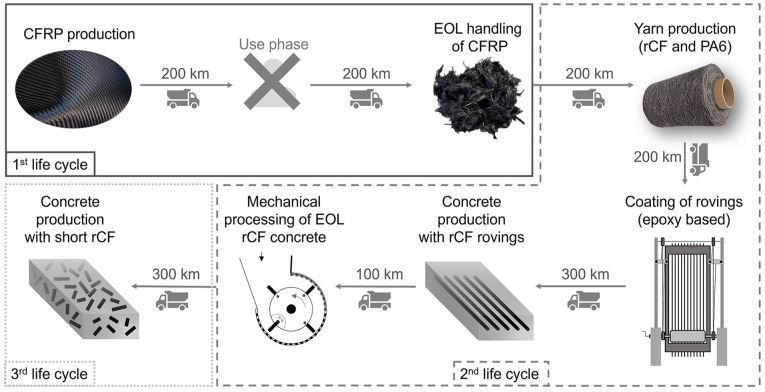
System boundaries and processes covered for each life cycle.

The research questions are mentioned previously in section ‘Introduction’ and an overview of the studied system is shown in Figure 1, covering the following life cycle stages:

→ First life cycle: CFs are used in CFRP production, transported to user, transported from user to EOL handling and recycled by means of pyrolysis.
→ Second life cycle: rCF are used together with polycaprolactam (PA6) in yarn production through spinning process, epoxy-based rovings are produced using rCF yarn, the rCF rovings are used in CFRC production and mechanically processed at the EOL.
→ Third life cycle: short rCF, that were separated from second life cycle, are transported, and used in concrete production. Fine and composite fractions after comminution process were assumed to be landfilled. The mineral fraction is assumed to be further used and taken as burden-free, as the focus in on rCFs.

The LCA model for the mechanical processing was modelled based on the average mass fraction of six machine settings, for the first part of the LCA, where three life cycles were assessed. A simplified LCA only focusing on the mechanical processing and aiming to compare different machine settings were performed separately under the techno-environmental assessment.

The FU of the study is selected as a 525 cm^3^ concrete block involving 6 g of short rCF with a concrete mix as shown in Supplemental Table S2 and aimed to be used for non-structural applications. The environmental impacts for each life cycle are presented based on the set FU; however, it is worth to mention that the environmental impacts of each life cycle should not be compared between them, as they cover different product groups and system boundaries.

In the comparative LCA between rCF and virgin CF, a FU of 1 kg CF was used. The system boundaries for the comparative LCA were set from raw material extraction to CF production for the virgin CF, and from transport of EOL CFRP to the pyrolysis plant to the rCF production for the rCF.

In order the answer the RQ5, a simplified LCA focusing only on the comminution process was performed to compare different machine settings from a techno-environmental perspective. The comparative LCA was modelled by considering only the percent mass of each fraction in the output after comminution. The electricity consumption for each machine setting was not included in the comparative LCA as it was not possible to measure the specific electricity consumption for each machine setting, and the difference in the electricity consumption was assumed to be minimal and therefore not included in the system boundaries. Similarly, the manual sorting process was not considered for the comparative LCA as it is difficult to be quantified and it was rather similar for all machine settings. The FU was set as 6 g of separated rCF after the comminution to be used as short rCFs in concrete production. The reference flows corresponding to the set FU were calculated based on the average percent mass for each output fraction (separated rCF, mineral, composite and fine), which are based on equations (1) and (2). In the base scenario, the composite and fine fractions were assumed to be landfilled, and the mineral fraction was taken as burden-free, assuming to be further used. However, and additional scenario that considers not only the composite and fine, but also the mineral fraction within the rest to be landfilled was performed.

#### Life cycle inventory

The background data were taken from ecoinvent v3.9.1 cut-off database and the studied system was modelled using Umberto 11 software (version 11.9.1) following an attributional LCA approach. The detailed list of the ecoinvent processes and literature-based assumptions that were used for LCA model can be found in the supplemental document-section ‘Life cycle inventory’.

The assumed transport distances are shown in Figure 1, and the transport distance assumptions were made based on Meng et al. (2018) for the first life cycle and the transport of rCF from pyrolysis. For the transport distance to the concrete production (both for rCF rovings and short rCFs), a distance of 300 km is assumed, as it is not a common application.

#### Life cycle impact assessment

LCIA was performed using Environmental Footprint (EF) 3.0 impact method. EF is developed by the European Commission as recommendations for impact categories and models for Product and Organisation Environmental Footprint (PEF/OEF) (Sala et al., 2019). In this study, the results interpretation was done on a mid-point level covering all the impact categories. However, for detailed assessments and sensitivity analysis, a specific focus was placed on global warming potential (GWP).

#### Sensitivity analysis

A sensitivity analysis is conducted using the sensitivity ratio (SR), see equation (4), as described by Bisinella et al. (2016).

(4) $${\mathrm{SR}}_{i}^{j}=\frac{{\left(\frac{\Delta \;result}{initial\;result}\right)}^{j}}{{\left(\frac{\Delta \;parameter}{initial\;parameter}\right)}_{i}}\approx \frac{\partial z_{j}}{\partial x_{i}}\frac{x_{i}}{z_{j}}$$

where *i* = 1, . . ., *n* tested parameters in the model and *j* = 1, . . ., *m* impact categories in the characterisation method selected for the calculation of the impacts.

Transport distances are included in the sensitivity analysis as transport distances were mainly assumption-based values. In addition, the electricity consumption for the mechanical processing is based on lab-scale application and the measured value is included in the sensitivity analysis, as the real application value may differ.

## Results and discussion

### Mechanical processing

**Figure 2.**
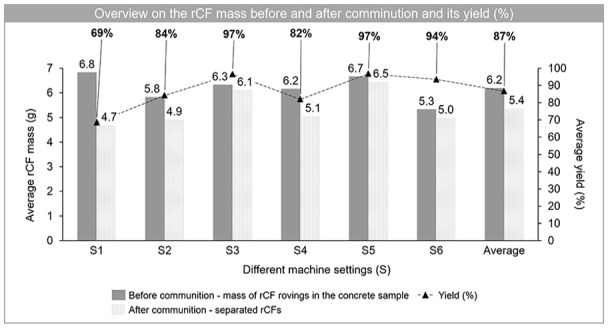
An overview of the mass (g) of the rCF rovings in concrete samples and after the comminution process as separated rCFs is shown on the left side of the *y*-axis. The mass values are given as the average of three repetitions for each machine setting. The rCF yield (%) calculated based on equation (3) is shown on the right side of the *y*-axis.

The average yield (%) for rCFs, which were calculated on the basis of equation (3), varies between 69% and 97% depending on the machine setting. An overview of the average yield values (%) for each setting is shown in Figure 2, which represents the separation potential of rCFs. It should be noted that the yield values include three repetitions for each machine setting and are therefore given as an average yield. In addition, an overview on the average mass of rCFs that were used as rovings in concrete samples and mass of separated rCFs after comminution are presented in Figure 2, in which again an average value of three repetitions for each machine setting is presented. The highest average yield (%) is observed in machine settings S3 and S5, where 97% of the input rCFs rovings were liberated from the concrete samples through the comminution process.

In addition to CF yield (%), two further data analyses were performed on mechanical processing, based on (1) wt.-% of four different fractions: rCF, mineral, composite and fine fractions, and (2) length of separated rCFs.

**Figure 3.**
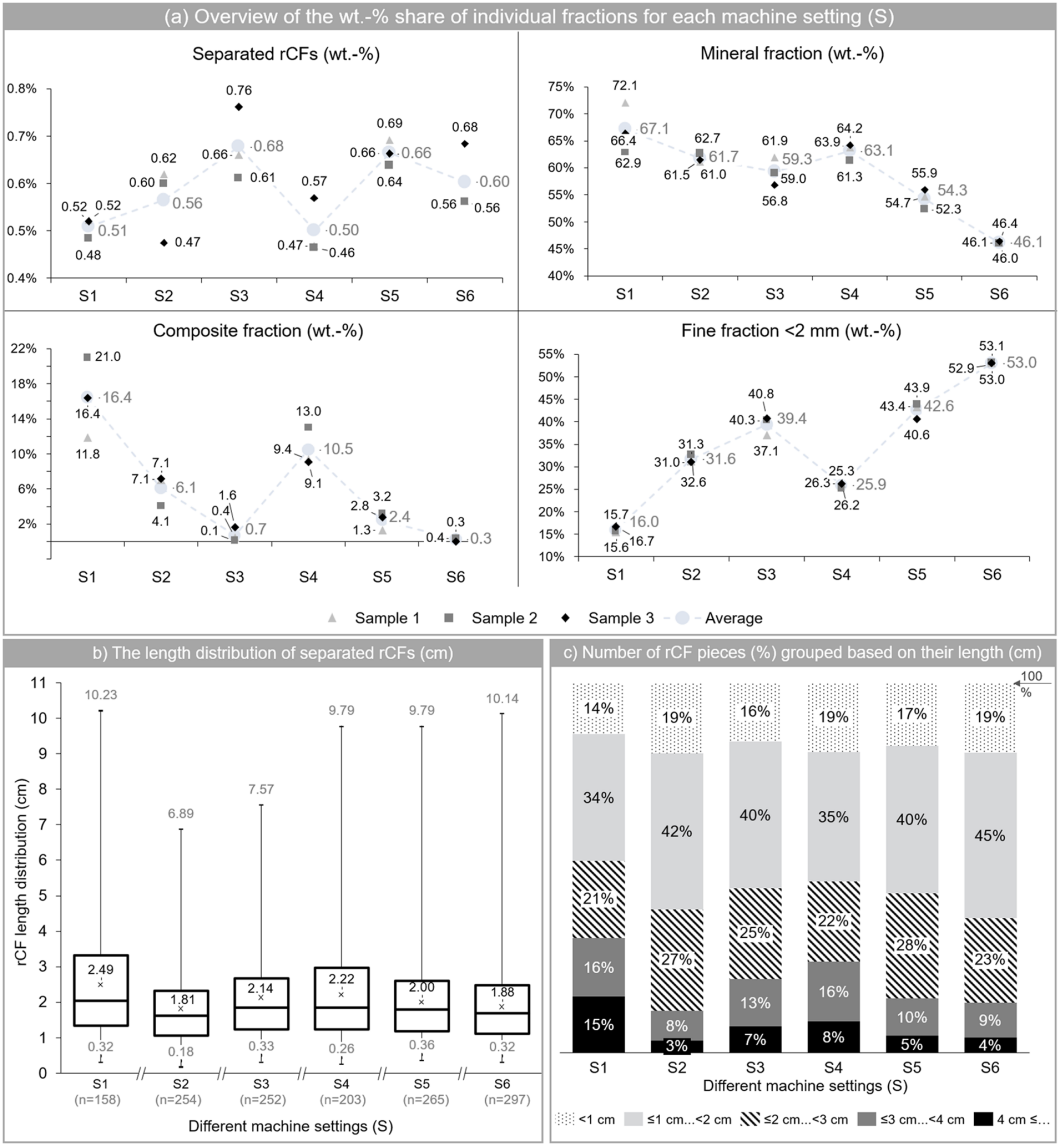
(a) Percent division by weight of four different fractions (CF, mineral, composite and fines <2 mm) after comminution process, (b) the length distribution of CFs in cm and (c) the number of CF pieces, presented as share and grouped based on their length range.

As shown in Figure 3, the maximum weight fraction of rCFs after comminution was achieved in S3 and S5, with 0.68 and 0.66 wt.-% of the total output, respectively. These two machine settings also had the lowest wt.-% of the composite fraction in the output, with a value of 0.7 wt.-% for S3 and 2.4 wt.-% for S5. At the same time, however, in these machine settings, relatively high percentage of fine fraction (<2 mm) was also observed with a value of 39.4 wt.-% in S3 and 42.6 wt.-% in S5. As further separation of rCF and mineral fractions within the fine fraction is very difficult; thus, S3 and S5 could be disadvantageous due to the increased proportion of the fine fraction. One the other hand, the composite fraction, which is rather low in S3 and S5 compared to the other machine settings, is also problematic in terms of further utilisation. Thus, when the fine and composite fractions are summed up as residual fraction that cannot be further utilised, then S3 becomes the most favourable option, followed by S5.

When it comes to the length of rCFs, S1 had the highest average length and highest percentage of rCFs with a length of equal and greater than 4 cm (15% of total number of the sorted rCFs). For all the machine settings, the majority of rCFs, with a share of about 35% of the total number of rCF pieces, ended up in the second rage (⩽1 cm. . .<2 cm) and this was followed by the third range (⩽2 cm. . .<3 cm), which covered more than 20% of the total number of rCF pieces. As shown in part (b) of Figure 3, S1 ended up with the highest average rCF length, followed by S4 and S3.

Although we tried to be as precise as possible during the mechanical processing experiments, it is possible that some errors may occur in the weight measurement of each fraction. This is especially true given the multiple trials conducted and the fact that the collection of crushed samples from the hammer mill was done manually, potentially resulting in losses or the inclusion of material from previous batches. Furthermore, it was not possible to measure dust production during the experiment, which is a critical factor to consider.

### LCA interpretation

#### Contribution analysis

**Figure 4.**
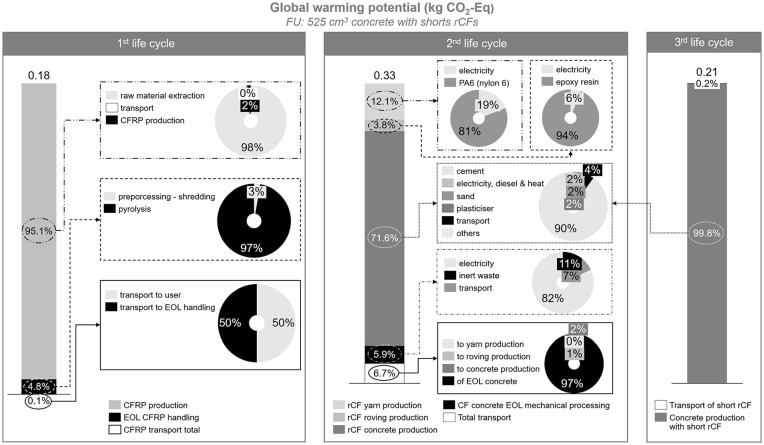
GWP overview of first, second and third life cycles. The results are shown per FU: 525 cm^3^ of concrete produced with 6 g of short rCFs (third product).

The results are presented based on the FU set: 525 cm^3^ concrete sample containing short rCFs with a mass of 6 g. For each life cycle, a contribution analysis on GWP is shown in Figure 4. Within the first life cycle, the CFRP production accounts for more than 95% of total GWP, followed by the pyrolysis of EOL CFRP with a value of 4.8% and transport with 0.2%. The GWP of pyrolysis process, also covering the transportation and pre-processing, was calculated as 6.6 kg CO_2_-Eq per kg rCF produced, which is close to the value of 5.4 kg CO_2_-Eq reported by Witik et al. (2013). It is worth nothing that a one-to-one comparison is not possible as the databases, software and LCIA methods used for the studies are different. In addition, the transport distances assumed in the studies differ. In the second life cycle, the concrete production accounts for more than 71% of total GWP, in which cement production has a share of 90%. The second highest contributor to GWP in the second life cycle is the yarn production, in which PA6 production causes around 81% of the total GWP impact. The transport process is the third contributor for the second life cycle and transport of EOL concrete to mechanical recycling accounts for 97% of the total GWP that was caused by the transport processes. Similar to the second life cycle, the main contributor for the third life cycle is the concrete production, accounting for 99.8% of the total GWP. The overview of results shown in Figure 4 should be interpreted with caution, as a comparison between the life cycles is not possible, since these three life cycles refer to three different products. This overview involves information on GWP for each life cycle and contribution analysis within each life cycle. As mentioned previously, the results are presented based on the FU of 525 cm^3^ concrete sample containing short rCFs with a mass of 6 g, which is the product of third life cycle. The reference flows that correspond to the selected FU for the second life cycle is the rCF concrete sample with a volume of 586.81 cm^3^ involving 7 g of rovings and for the first life cycle is then 2 g of rCFs that are produced through pyrolysis.

A summary of the results for each life cycle, including contribution analysis for all impact categories is presented in Supplemental Table S4. Even though the same trend was observed for all the impact categories, and discussed previously for GWP, a slight difference was observed in the share of the environmental impacts for each process. For instance, the impact of the pyrolysis process was much higher for ionising radiation and ozone layer depletion, with a share of 13% and 7%, respectively. The main reason behind the increased impacts in these two impact categories is due to the incinerated portion of CFRP during pyrolysis. Considering the water scarcity impact, yarn production accounted for 24% of the total impact in the second life cycle, which was 12% in GWP. Here, the increased share in water scarcity is mainly caused by PA6 production. The EOL handling of concrete with rCF rovings had the highest contribution in ionising radiation and land use and land use change impacts, with a share of 26% and 25%, respectively. This is due to the rest fraction (composite and fines <2 mm) which was assumed to be landfilled.

Another important point that should be considered during the result interpretation is the system approach. In this study, we followed cut-off approach and allocated the impact of recycling to the first product system and considered the recycled product burden-free. For instance, the impacts of pyrolysis were allocated to 100% to CFRP EOL handling, and for the second life cycle, the rCFs from pyrolysis were assumed to have zero impact. A similar approach was also applied between the second and third life cycle in the allocation of the impacts between mechanical processing of rCF concrete in the second life cycle and the use of short fibres in concrete within the third life cycle. In LCA, focusing on recycling systems, the selected allocation approach can have a big impact on the results; and thus, plays an important role. There are various allocation approaches, such as simple cut-off, 50/50 method, market price-based substitution, the circular footprint formula (CFF) and others (Ekvall et al., 2020). Each approach has its own advantages and disadvantages. For instance, considering the CE, market price-based substitution and CFF consider the quality aspect, but they are rather complex and require more data compared to simple cut-off. In this study, the main reason for applying the simple cut-off approach was due to the scope of the study and limited data availability on quality of the CFs. At this point, however, modelling with different allocation approaches is relevant and will be interesting to further evaluate the differences between the various system approaches.

#### Comparison of rCF and virgin CF

**Figure 5.**
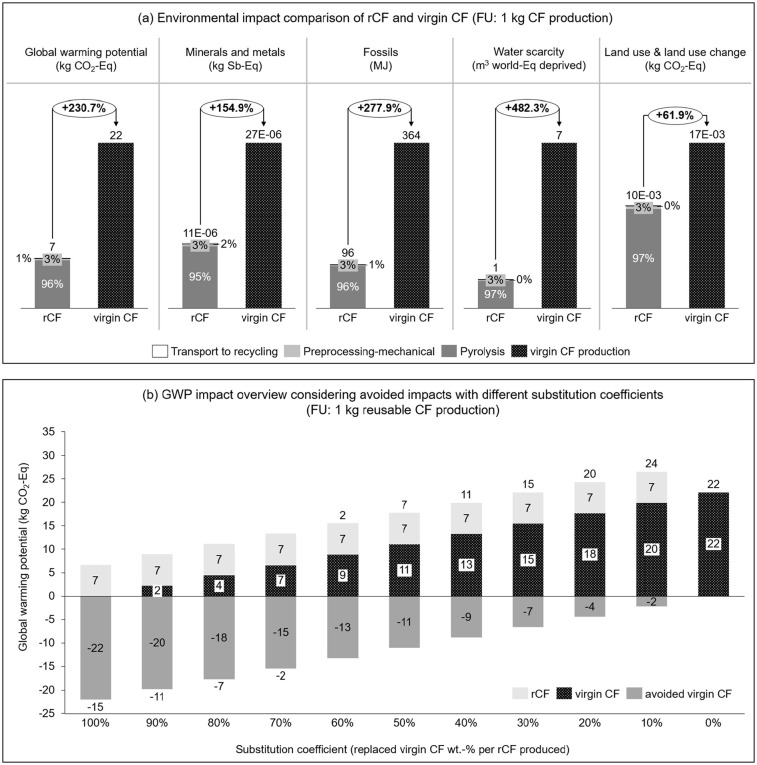
Comparison of virgin vs. rCF production. (a) Overview on different environmental impact categories, no avoided impacts are considered. (b) GWP comparison, considering avoided impacts with different substitution coefficients.

A comparative LCA on the production of rCF through pyrolysis and virgin CF was conducted. The GWP of virgin CF production was calculated as 22 kg CO_2_-Eq per kg fibre, which is higher than what is reported as 17.2 CO_2_-Eq by Karuppannan Gopalraj et al. (2021). The main difference may be due to the use of market processes, but a one-to-one comparison is not possible due to the use of different databases, software and LCIA methods. The comparative LCA results show that rCF has great environmental savings over virgin CFs, up to 482% savings in water scarcity impact (see Figure 5). For GWP, the difference is 230%, and the lowest difference is observed for land use and land use change with a value of 62%. In this comparison, it was assumed that the quality of recycled and new CF is the same, as it was not possible to quantify the quality of rCF compared to new CF. Therefore, this result should be interpreted with caution. In addition, in the first comparison, in the part (a) of Figure 5, no avoided impacts through the rCF production were considered. In the part (b) of Figure 5, avoided virgin CF through the rCF production as well as the substitution ratio for the virgin CF were considered. Here, it was assumed that 1 kg of rCF will be produced, but it can substitute different wt.-% of virgin CF. The results show that the rCF should at least replace 20 wt.-% of virgin CF, so that it still has lower GWP compared to virgin CF. It is important to note that the calculation is done considering the usable rCF, meaning that if rCF can only substitute 10 wt.-% of the virgin CF, the environmental impact of recycling (pyrolysis process) for 100 wt.-% of rCF was still accounted fully.

The inclusion of recycled material quality in LCA is essential to differentiate downcycling and recycling (Di Maria et al., 2018); however, there is a limited number of studies that considered the quality aspect within LCA (Bayram and Greiff, 2023; Di Maria et al., 2018; Laurent et al., 2014). Even though the importance of quality consideration in recycling system is of discussion, quality is rather undefined, and the quantification of quality can be data and time intensive as it is specific to the case studied (Eriksen et al., 2019; Helbig et al., 2022; Tonini et al., 2022). Therefore, further research focusing on quality quantification and a standardised framework for it would be useful.

#### Sensitivity analysis

Due to the lack of information, some of the transport distances are based on assumptions and were therefore included in the sensitivity analysis. The assumed transport distances were doubled, and the SR values were observed to be relatively low varying between 4.30E-04 and 6.46E-02, for transport of CFRP from and to the user and EOL concrete with rCF rovings, respectively. As the mass of the concrete with rCF rovings is the highest, the change in the transport distance has the highest impact compared to other transport distances. The second highest change was observed for the transport of short rCFs with a SR value of 1.62E-03. Based on the results, it can be said that the transport assumptions rather cause slight difference; except for the transport of EOL concrete with rCF rovings. Another parameter that was included in the sensitivity analysis is the mechanical processing of the concrete with rCF rovings, as the energy demand during mechanical processing was measured in lab scale with a possible error margin, and it does not represent the industrial scale. In addition, the energy demand of sieving and hand-sorting that were applied after comminution was not included in the calculation. Thus, within the sensitivity analysis, the energy demand for mechanical processing increased by 4 times, which ended up with a SR value of 4.80E-02, and the process contribution within the second life cycle increased from 6% to 21%, which is relatively high. In view of these results, it is recommended to model the mechanical processing in more detail and to use industrial data if available. The SR values and the change in process contribution for the transport distance and energy demand for mechanical processing are presented in Supplemental Table S5.

### Techno-environmental assessment

**Figure 6.**
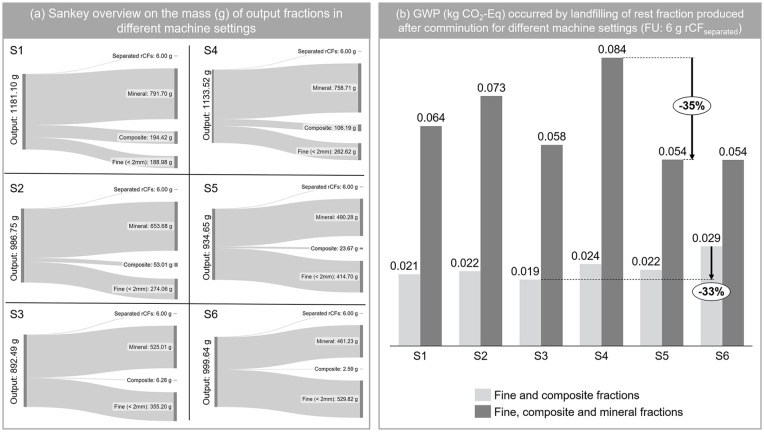
Summary on the techno-environmental assessment: (a) Sankey diagram illustrating the mass of output fractions generated by different machine settings, normalised to the FU of 6 g of separated rCFs; and (b) an overview of the GWP for each machine setting, considering two scenarios with and without the mineral fraction within the rest portion to be landfilled.

A simplified LCA was performed to compare the environmental impact of different machine settings and enable techno-economic analysis considering the mechanical processing results (Section ‘Mechanical processing’). While doing this, as previously mentioned, only the rest portion in the output that cannot be further utilised considered. In order to enable the comparison, the reference flows for each output fraction (mineral, composite and fine) were calculated based on the set FU, 6 g of rCF separated. The mass flows used for the comparative LCA is presented in part (a) of Figure 6. The lowest percent mass of the rest fraction (composite and fine) in the output was achieved in S3; thus, having the lowest GWP, when the mineral fraction was not considered to be landfilled. However, when the mineral fraction was assumed to be landfilled, in addition to fine and composite, then, the GWP of S5 and S6 ends up with the lowest impact.

When considering the technical assessment parameters of rCF yield (%) and length (cm) along with the comparative LCA results, S3 is the most favourable option when mineral fraction is burden-free (yield of 97%, rCF length over 2 mm at 45%, and GWP of 0.019 kg CO_2_-Eq). However, when mineral fraction is considered as landfilled, S5 becomes the most favourable option with a yield of 97%, rCF length over 2 mm at 43% and GWP of 0.054 kg CO_2_-Eq. The difference between two scenarios is the same for all the impact categories, as the model only involves one databank process, ‘market for inert waste (Europe without Switzerland)’. The LCA result for all other impact categories can be seen in Supplemental Table S6.

It is worth mentioning that handling and utilisation potential for each output fraction is based on assumptions, as practical examples are limited. Therefore, scenarios for further use options for the mineral fraction were not included, which is an essential point that can have a significant impact on LCA results. Moreover, it should be noted that the technology readiness level (TRL) for mechanical recycling and the use of CF as short fibres on a laboratory scale reflects a level between basic and applied research that can be classified between TRL 1 and 2. Thus, a prospective LCA could be further performed to estimate future potential.

## Conclusion

In this study, the use of rCF as rovings in concrete production, as well as its EOL handling through mechanical processing were evaluated. Specifically, the potential for separating rCF from concrete and reusing it in concrete production as short rCFs was assessed. Based on the results of the EOL handling experiments, we found that rCF rovings were able to be separated from concrete with an average yield of 87% and a maximum rate of 97%. To optimise the separation process, different settings on the hammer mill machine was tested, and it was found that S3 (1500 min^−1^ with a grate size of 40 mm) and S5 (2000 min^−1^ with a grate size of 60 mm) produced the highest yield (%) for rCF rovings. However, these settings also generated a significant mass of fine fractions (<2 mm) that could not be further separated. Additionally, we assessed the length of the separated rCFs and found that S1 (1500 min^−1^ with no grate) had the highest proportion (51%) of rCFs with a length above 2 cm and 15% above 4 cm. Based on the technical assessment, it can be concluded that different machine settings can be selected depending on the specific aim. For example, if the aim is to achieve the highest possible separation rate of rCF, then S3 (1500 min^−1^ with a grate size of 40 mm) and S5 (2000 min^−1^ with a grate size of 60 mm) are the best options. On the other hand, if the goal is to achieve the longest possible length of rCF with the lowest proportion of fine fraction, then S1 (1500 min^−1^ with no grate) is a better choice. It is worth mentioning that errors may occur during the mechanical processing experiment, primarily due to the potential mixing of the current batch with a small remainder of the previous batch that was left in the hammer mill. Additionally, there is a lack of data regarding dust measurements, which is another important factor to consider.

The LCA results revealed that the CFRP production (over 95% in GWP) within first life cycle and concrete production processes in second and third life cycles are the main contributors for the total environmental impacts, considering all the environmental impact categories. While conducting the LCA, simple cut-off approach was followed; however, different impact allocation approaches (quality adjusted 50/50 or CFF) between two life cycles in recycling systems will be convenient to be applied. In this study, we did not include comparison of different allocation approaches, as required data, especially in the quality aspect was not available. Still, it will be a relevant aspect to be further assessed.

A comparative LCA on virgin CF and rCF production was performed and observed that production of 1 kg of rCF causes 6.7 kg CO_2_-Eq and this value goes up to 22 kg CO_2_-Eq for virgin CF. However, no avoided impacts nor quality difference between the rCF and virgin CF were considered. An assumption-based scenario analysis for the comparison of virgin CF and rCF considering various substitution coefficient and avoided virgin CF was also performed. The results revealed that the substitution coefficient should be at least 20% for rCF to have a lower GWP compared to virgin CF. Further assessment on quantification of the quality of rCF compared to virgin CF based on the real-life application and market situation will be highly relevant. From an environmental point of view, further studies assessing different allocation approaches between two life cycles of recycling systems, quality quantification and its inclusion in LCA will be relevant.

Finally, a techno-environmental assessment was performed, where different machine settings were compared based on rCF yield, the length of separated rCF and environmental impact of each machine setting which is calculated based on the mass of rest fraction that cannot be further utilised. The results showed that S3 (1500 min^−1^ with a grate size of 40 mm) becomes the most favourable option if the rest fraction covers only fine and composite; however, if mineral fraction is also included in the rest portion, then S5 (2000 min^−1^ with a grate size of 60 mm) is more advantageous considering both technical and environmental aspects.

## Supplemental Material

sj-docx-1-wmr-10.1177_0734242X241237197 – Supplemental material for Recycling potential of carbon fibres in the construction industry: From a technical and ecological perspective Supplemental material, sj-docx-1-wmr-10.1177_0734242X241237197 for Recycling potential of carbon fibres in the construction industry: From a technical and ecological perspective by Berfin Bayram, Vanessa Overhage, Marco Löwen, Katharina Terörde, Karoline Raulf, Kathrin Greiff and Thomas Gries in Waste Management & Research
