# Supplementary material for: Recycling potential of carbon fibres in the construction industry: From a technical and ecological perspective
Source: Waste Manag Res. 2024 Apr 17;42(9):726–37. doi: 10.1177/0734242X241237197 (PMC11370193; doi:10.1177/0734242X241237197)
Supplement: sj-docx-1-wmr-10.1177_0734242X241237197 – Supplemental material for Recycling potential of carbon fibres in the construction industry: From a technical and ecological perspective [file sj-docx-1-wmr-10.1177_0734242X241237197.docx]

**Supplementary Document**

**Recycling Potential of Carbon Fibres in the Construction Industry:**

***from a technical and ecological perspective***

**Berfin Bayram^a*^, Vanessa Overhage^b^,** Marco Löwen^c^, Katharina Terörde^c^**, Karoline Raulf^a^, Kathrin Greiff^a^, Thomas Gries^b^**

^*^ Corresponding author

**^a)^ RWTH Aachen University, Department of Anthropogenic Material Cycles (ANTS), Aachen, Germany**

**^b)^ RWTH Aachen University, Institut fuer Textiltechnik (ITA), Aachen, Germany**

^c)^ **RWTH Aachen University, Aachen, Germany**

# Glossary

Table S1. The glossary of terms used for the end-of-life handling of carbon fibre composites (CF).

| Term | Description |
| --- | --- |
| Mechanical recycling | Processing of CF composites using mechanical forces such as crushing, grinding, milling, and shredding to achieve the particle size reduction. Furthermore, different classification and sorting techniques can be applied without changing the chemical composition of materials (Borjan et al., 2021; Karuppannan Gopalraj et al., 2021). |
| Thermal recycling | Processing of CF using heat, resulting in the combustion of insignificant volatiles while retaining valuable CF. There are different types of thermal recycling processes such as pyrolysis and fluidized bed (Borjan et al., 2021; Karuppannan Gopalraj et al., 2021). |
| Chemical recycling | Processing of CF composites by dissolving them in a chemical solution to enable resin degradation using solvents (solvolysis) or water (hydrolysis) (Borjan et al., 2021; Karuppannan Gopalraj et al., 2021). |
| Pyrolysis | A thermal recycling process that enables the decomposition of polymers at high temperatures between 350/400 and 700 degrees in the absence of oxygen and an inert atmosphere. Pyrolysis not only recovers the fibres and fillers, but also produces oil and gas (Borjan et al., 2021; Karuppannan Gopalraj et al., 2021). |
| Solvolysis | A chemical recycling process in which solvents are used to break the matrix bond under a certain pressure and temperature to reclaim the CFs (Borjan et al., 2021; Karuppannan Gopalraj et al., 2021; Wei and Hadigheh, 2022). |
| Degradable resin | This term is used by Wang et al. (2023) referring to resins that can be degraded by the introduction of unstable chemical bonds or by cleaving some of the chemical bonds, which leads to the recovery of CFs. The term is used within the context of electrochemical recycling (Wang et al., 2023). |
| Thermolysis | The term is used instead of pyrolysis (López et al., 2013; Naqvi et al., 2018) |
| Gasification | An oxidative treatment applied after pyrolysis to clean the fibre surface and remove amorphous pyrolytic carbon and some resin residues without damaging the CFs (López et al., 2013; Mazzocchetti et al., 2018; Naqvi et al., 2018). |
| Catalytic pyrolysis | A process based on chemical degradation of CF composite by thermal energy in the presence of a catalyst. The term is used by Wei and Hadigheh (2022) and aluminium chloride was used as catalyst in the study. |
| Oxidation | According to Wei and Hadigheh (2022), oxidation is defined as a thermal recycling method where the reaction occurs in the presence of the air, unlike pyrolysis. |
| Grinding | A technique used in mechanical processing to reduce the particle size and thus also the CF length (Vo Dong et al., 2018). |
| Microwave treatment | According to Vo Dong et al. (2018), it is a thermal technique that enables matrix recovery with less energy usage compared to pyrolysis. |
| Supercritical fluids  (e.g. supercritical water) | Supercritical fluids are used as solvents in chemical recycling, where liquids are used at temperatures and pressures (generally just above the critical point). Supercritical fluids have combined properties, i.e. they can penetrate porous solids and dissolve organic materials, while they are still relatively innocuous under atmospheric conditions. As one of the supercritical fluids, water has the advantage of being cost-effective and low-risk compared to organic solvents (Pimenta and Pinho, 2011; Vo Dong et al., 2018). |

# Mechanical processing:

The hammer mill used in comminution experiments are illustrated in Figure S1.


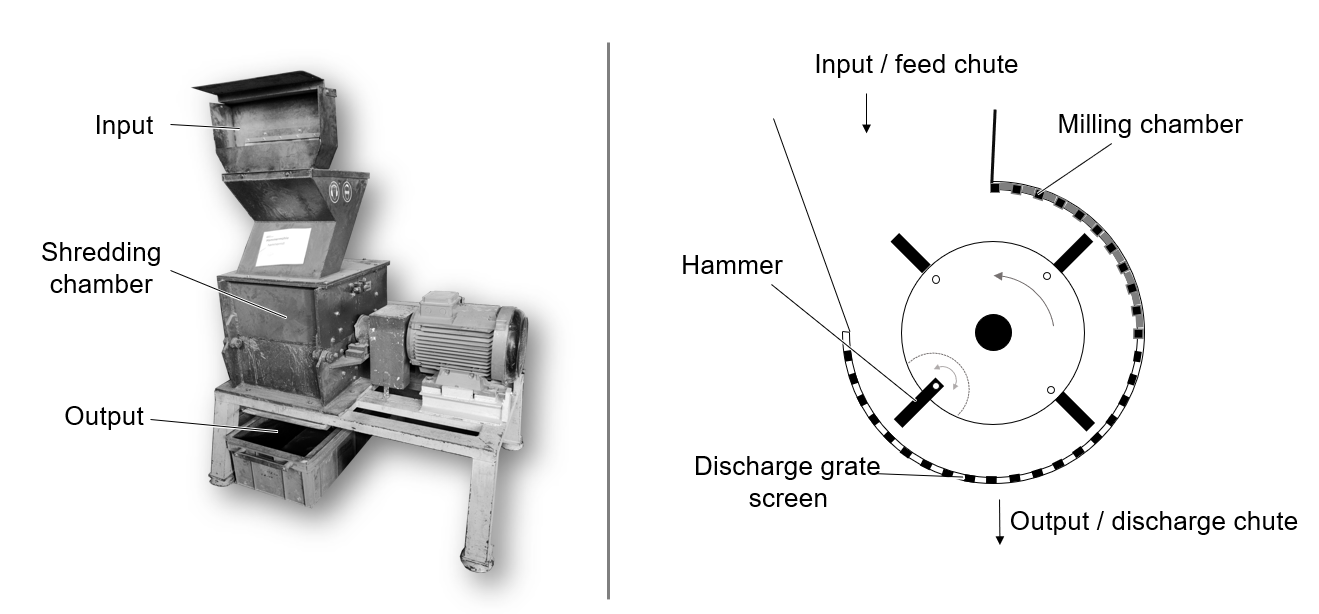


Figure S1. Illustration of hammer mill used in the experiment.

# Concrete production:

The concrete mix used for the concrete production is presented in Table S2.

Table S2. Concrete mix used, based on ITA, RWTH Aachen

| Component | Unit | Quantity [g] |
| --- | --- | --- |
| Cement CEM l 42.5 R | g/dm^3^ | 490 |
| Fly ash | g/dm^3^ | 175 |
| Silica fume | g/dm^3^ | 35 |
| Quartz powder | g/dm^3^ | 500 |
| Sand 0.2 - 0.6 mm | g/dm^3^ | 713 |
| Water | g/dm^3^ | 280 |
| Superplasticizer | g/dm^3^ | 7 |

# Life cycle inventory:

The following ecoinvent activities were used for the background processes:

- CFRP production: “carbon fibre reinforced plastic, injection moulded [GLO]”
- Transport processes: “market for transport, freight, lorry 16-32 metric ton, EURO5 [RER]”
- Electricity: “market for electricity, medium voltage [DE]”
- Pyrolysis: modelled based on Vo Dong et al. (2018), in which energy input was taken as 30 MJ/kg and outputs consist of 65% CF and 35% matrix. For the incineration of the matrix, the ecoinvent activity “treatment of municipal solid waste, incineration [DE]” was used.
- Production of rCF yarn through spinning: For the yarn production from rCFs, the spinning process was modelled according to Goyal and Nayak (2020), in which 72% of PA6 and 28% of rCF by weight are used and 3.5 kWh electricity per kg yarn produced is required.
- Production of rCF rovings: the electricity requirement for the textile production was modelled according to Hohmann (2019), in which 0.052 kWh per m^2^ textile reinforcement was given.
- For the rCF textile concrete production, concrete mix from ITA was used for the main inputs. For the background processes (electricity, wastewater, waste concrete, concrete mixing factory etc.) of concrete production, ecoinvent activity “concrete production, 40MPa, for civil engineering, with cement, Portland [ROW]” was used and adjusted to DE energy mix.
- For the mechanical processing with hammer mill, electricity demand was measured during the experiment, with an average of 0.03 kWh/kg. For the electricity, ecoinvent activity “market for electricity, medium voltage [DE]” was used.
- Virgin CF production is modelled based on Karuppannan Gopalraj et al. (2021) and the ecoinvent activities that were used for the modelling are presented in the supplementary document.
- For the EOL handling of rest fraction after comminution, “market for inert waste [Europe without Switzerland]” was used.

Life cycle inventory (LCI) used for modelling virgin CFs and used ecoinvent processes are presented in Table S3.

Table S3. Life cycle inventory used for virgin CF production modelling, according to Karuppannan Gopalraj et al. (2021).

| Inputs | Amount | Unit | Ecoinvent process used |
| --- | --- | --- | --- |
| Amonium bicarbonate | 0.02 | kg | market for ammonium bicarbonate (RER) |
| Epoxy resin | 0.01 | kg | market for epoxy resin, liquid (RER) |
| Polyacrylonitrile fibers | 1.89 | kg | acrylonitrile-butadiene-styrene copolymer production (RER) |
| Polydimethylsolixane | 0.01 | kg | market for polydimethylsiloxane (GLO) |
| Potassium permanganate | 0.1 | kg | market for potassium permanganate (GLO) |
| Sulfuric acid | 0.02 | kg | sulfuric acid production (RER) |
| Water | 2.77 | 1 | market for tap water (Europe without Switzerland) |
| Electricity | 20.2 | kWh | market for electricity, medium voltage (DE) |
| Heat | 98.4 | MJ | heat and power co-generation, natural gas, combined cycle power plant, 400MW electrical (DE) |
| Outputs | **Amount** | **Unit** |  |
| Carbon fibers | 1 | kg |  |
| Carbon dioxide | 0.63 | kg | Carbon dioxide, fossil (emissions to air, unspecified) |
| Nitrogen monoxide | 0.33 | kg | Nitrogen oxides (emissions to air, unspecified) |
| Nitrogen dioxide | 0.66 | kg | Nitrogen dioxide (emissions to water, surface water) |

# Life cycle assessment results

For the LCA covering three life cycles of CFS, a summary on the LCA results covering all life cycles and contribution analysis within each life cycle for all impact categories are presented in Table S4.

Table S4. An overview on total environmental impacts and contribution analysis for 1^st^, 2^nd^ and 3^rd^ life cycles, covering all impact categories.

| Life cycle | Carcinogenic effects  CTUh | Climate change  kg CO2-Eq | Fossils  MJ | Freshwater ecotoxicity  CTUe | Freshwater eutrophication  kg P-Eq | Ionizing radiation  kBq U235-Eq | Land use  points | Land use and  land use change  kg CO2-Eq | Marine eutrophication  kg N-Eq | Minerals and metals  kg Sb-Eq | Non-carcinogenic effects  CTUh | Ozone layer depletion  kg CFC-11-Eq | Photochemical ozone creation  kg NMVOC-Eq | Respiratory effects, inorganics  Disease incidences | Terrestrial eutrophication  mol N-Eq | Water scarcity  m^3^ world-Eq deprived |
| --- | --- | --- | --- | --- | --- | --- | --- | --- | --- | --- | --- | --- | --- | --- | --- | --- |
| Total 1^st^ life cycle | **4.97E-11** | **1.79E-01** | **2.13** | **4.15** | **6.87E-05** | **1.47E-02** | **5.89E-01** | **2.14E-04** | **1.59E-04** | **5.18E-07** | **1.93E-09** | **1.02E-09** | **5.01E-04** | **1.28E-08** | **1.61E-03** | **3.86E-02** |
| Raw material production for CFRP | 93.89% | 93.69% | 92.20% | 96.85% | 81.73% | 84.39% | 93.91% | 91.90% | 94.85% | 96.47% | 94.58% | 87.38% | 95.91% | 98.49% | 95.87% | 93.38% |
| CFRP production | 1.85% | 1.45% | 1.91% | 1.16% | 1.47% | 2.60% | 2.95% | 1.91% | 1.41% | 0.82% | 1.25% | 5.04% | 1.61% | 0.80% | 1.40% | 2.68% |
| Transport of CFRP to user | 0.07% | 0.04% | 0.05% | 0.03% | 0.01% | 0.01% | 0.11% | 0.02% | 0.05% | 0.05% | 0.05% | 0.16% | 0.07% | 0.05% | 0.06% | 0.01% |
| Transport EOL CFRP for handling | 0.07% | 0.04% | 0.05% | 0.03% | 0.01% | 0.01% | 0.11% | 0.02% | 0.05% | 0.05% | 0.05% | 0.16% | 0.07% | 0.05% | 0.06% | 0.01% |
| EOL CFRP pre-processing | 0.11% | 0.13% | 0.16% | 0.05% | 0.47% | 0.37% | 0.08% | 0.17% | 0.10% | 0.07% | 0.11% | 0.20% | 0.06% | 0.02% | 0.07% | 0.11% |
| EOL CFRP pyrolysis | 4.01% | 4.64% | 5.63% | 1.89% | 16.32% | 12.62% | 2.84% | 5.98% | 3.54% | 2.54% | 3.95% | 7.06% | 2.27% | 0.60% | 2.54% | 3.80% |
| Total 2^nd^ life cycle | **8.59E-11** | **3.35E-01** | **2.56** | **3.29** | **7.05E-05** | **1.51E-02** | **1.19** | **1.15E-04** | **2.59E-04** | **1.32E-06** | **2.89E-09** | **3.87E-09** | **8.59E-04** | **9.22E-09** | **2.76E-03** | **4.72E-02** |
| Transport of rCF to yarn production | 0.03% | 0.01% | 0.03% | 0.02% | 0.00% | 0.01% | 0.03% | 0.02% | 0.02% | 0.01% | 0.02% | 0.03% | 0.03% | 0.04% | 0.02% | 0.01% |
| rCF yarn production | 4.76% | 12.13% | 19.56% | 2.92% | 16.46% | 12.01% | 1.48% | 10.92% | 12.85% | 17.73% | 4.37% | 2.21% | 12.29% | 15.62% | 11.38% | 23.92% |
| Transport for rCF roving production | 0.09% | 0.05% | 0.10% | 0.08% | 0.02% | 0.02% | 0.12% | 0.07% | 0.08% | 0.04% | 0.08% | 0.10% | 0.10% | 0.15% | 0.07% | 0.03% |
| rCF roving production | 16.06% | 3.77% | 9.61% | 16.95% | 6.17% | 7.00% | 2.56% | 10.00% | 3.93% | 9.05% | 6.59% | 38.81% | 5.01% | 5.36% | 3.79% | 14.19% |
| Transport for rCF concrete | 0.21% | 0.12% | 0.22% | 0.17% | 0.04% | 0.05% | 0.28% | 0.17% | 0.17% | 0.10% | 0.18% | 0.22% | 0.22% | 0.34% | 0.17% | 0.06% |
| rCF concrete production | 60.91% | 71.59% | 46.75% | 63.99% | 42.17% | 52.69% | 67.99% | 45.39% | 66.35% | 65.16% | 72.50% | 41.10% | 64.36% | 53.86% | 69.41% | 51.78% |
| Transport EOL rCF concrete | 11.17% | 6.46% | 11.74% | 9.24% | 2.09% | 2.65% | 14.94% | 8.93% | 9.12% | 5.25% | 9.65% | 11.84% | 11.93% | 18.16% | 9.05% | 3.12% |
| EOL rCF concrete mechanical processing | 6.79% | 5.86% | 11.99% | 6.62% | 33.06% | 25.58% | 12.59% | 24.50% | 7.48% | 2.66% | 6.60% | 5.70% | 6.07% | 6.48% | 6.12% | 6.89% |
| Total 3^rd^ life cycle | **4.69E-11** | **2.15E-01** | **1.08** | **1.89** | **2.66E-05** | **7.11E-03** | **7.26E-01** | **4.70E-05** | **1.54E-04** | **7.70E-07** | **1.88E-09** | **1.43E-09** | **4.96E-04** | **4.47E-09** | **1.72E-03** | **2.19E-02** |
| Transport of short rCFs | 0.33% | 0.16% | 0.45% | 0.26% | 0.09% | 0.09% | 0.39% | 0.35% | 0.25% | 0.14% | 0.24% | 0.52% | 0.33% | 0.60% | 0.23% | 0.11% |
| Concrete production with short rCFs | 99.67% | 99.84% | 99.55% | 99.74% | 99.91% | 99.91% | 99.61% | 99.65% | 99.75% | 99.86% | 99.76% | 99.48% | 99.67% | 99.40% | 99.77% | 99.89% |

The results overview of the sensitivity analysis is given in Table S5. The overview contains information on the sensitivity ratios (SR) and process contribution to the corresponding life cycle of base scenario and the additional scenario with the increased values.

Table S5. Result overview of sensitivity analysis.

| Life cycle | Process | Value base scenario | Increased value | SR | GWP share %  base scenario | GWP share %  increased value |
| --- | --- | --- | --- | --- | --- | --- |
| 1^st^ | Transport of CFRP to user | 200 km | 400 km | 4.30E-04 | 0.043% | 0.086% |
|  | Transport of EOL CFRP from user | 200 km | 400 km | 4.30E-04 | 0.043% | 0.086% |
| 2^nd^ | Transport of rCF to yarn production | 200 km | 400 km | 1.50E-04 | 0.015% | 0.030% |
|  | Transport of rovings to concrete production | 300 km | 600 km | 5.34E-04 | 0.053% | 0.107% |
|  | Transport of EOL concrete to mechanical processing | 100 km | 200 km | 6.46E-02 | 6.5% | 12% |
| 3^rd^ | Transport short rCF to concrete production | 200 km | 400 km | 1.62E-03 | 0.162% | 0.32% |
| 2^nd^ | Mechanical processing - electricity | 0.03 kWh/kg | 0.15 kWh/kg | 4.80E-02 | 6% | 21% |

LCA for the techno-environmental analysis was performed to compare different machine settings. As in the base scenario, it was assumed that the mineral fraction will be further used; thus, taken as burden-free by-product. In the base scenario, only fine and composite fractions were assumed to be landfilled. As no technical assessment for the further use option for by-products was performed, an additional scenario, considering mineral fraction to be also landfilled, in addition to fine and composite fractions, was modelled. The LCA results are presented in Table S6, covering all impact categories. As this comparative LCA only considers the landfilled fraction, which is taken from the ecoinvent (3.9.1 cut-off) database, “market for inert waste [Europe without Switzerland]”, the difference between the two scenarios within each machine setting is the same for all the impact categories.

Table S6. LCA result overview for different machine settings considering all the impact categories. The results are shown both for the base scenario, where fine and composite fractions were assumed to be landfilled, and mineral fraction was considered burden-free. The additional scenario, where all non-separated rCFs fractions, fine, composite and mineral, were assumed to be landfilled.

| **Machine Setting** | **Carcinogenic effects CTUh** | **Climate change**  **kg CO_2_-Eq** | **Fossils**  **MJ** | **Freshwater ecotoxicity**  **CTUe** | **Freshwater eutrophication**  **kg P-Eq** | **Ionizing radiation**  **kBq U235-Eq** | **Land use**  **points** | **Land use and**  **land use change**  **kg CO_2_-Eq** | **Marine eutrophication**  **kg N-Eq** | **Minerals and metals**  **kg Sb-Eq** | **Non-carcinogenic effects**  **CTUh** | **Ozone layer depletion**  **kg CFC-11-Eq** | **Photochemical ozone creation**  **kg NMVOC-Eq** | **Respiratory effects, inorganics**  **Disease incidences** | **Terrestrial eutrophication**  **mol N-Eq** | **Water scarcity**  **m^3^ world-Eq deprived** |
| --- | --- | --- | --- | --- | --- | --- | --- | --- | --- | --- | --- | --- | --- | --- | --- | --- |
| **Base scenario** | | | | | | | | | | | | | | | | |
| **S1** | 6.19E-12 | 2.09E-02 | 0.33 | 2.31E-01 | 2.48E-05 | 4.10E-03 | 1.59E-01 | 3.00E-05 | 2.06E-05 | 3.72E-08 | 2.03E-10 | 2.35E-10 | 5.55E-05 | 6.35E-10 | 1.79E-04 | 3.46E-03 |
| **S2** | 6.46E-12 | 2.17E-02 | 0.34 | 2.41E-01 | 2.58E-05 | 4.27E-03 | 1.66E-01 | 3.13E-05 | 2.14E-05 | 3.88E-08 | 2.11E-10 | 2.45E-10 | 5.78E-05 | 6.62E-10 | 1.87E-04 | 3.61E-03 |
| **S3** | 5.73E-12 | 1.93E-02 | 0.30 | 2.14E-01 | 2.29E-05 | 3.79E-03 | 1.47E-01 | 2.78E-05 | 1.90E-05 | 3.44E-08 | 1.88E-10 | 2.17E-10 | 5.13E-05 | 5.88E-10 | 1.66E-04 | 3.20E-03 |
| **S4** | 7.07E-12 | 2.38E-02 | 0.37 | 2.64E-01 | 2.83E-05 | 4.68E-03 | 1.82E-01 | 3.43E-05 | 2.35E-05 | 4.25E-08 | 2.32E-10 | 2.68E-10 | 6.33E-05 | 7.25E-10 | 2.05E-04 | 3.95E-03 |
| **S5** | 6.59E-12 | 2.22E-02 | 0.35 | 2.46E-01 | 2.64E-05 | 4.36E-03 | 1.69E-01 | 3.19E-05 | 2.19E-05 | 3.96E-08 | 2.16E-10 | 2.49E-10 | 5.90E-05 | 6.76E-10 | 1.91E-04 | 3.68E-03 |
| **S6** | 8.60E-12 | 2.90E-02 | 0.45 | 3.21E-01 | 3.44E-05 | 5.69E-03 | 2.21E-01 | 4.17E-05 | 2.86E-05 | 5.17E-08 | 2.82E-10 | 3.26E-10 | 7.70E-05 | 8.82E-10 | 2.49E-04 | 4.81E-03 |
| **Additional scenario** | | | | | | | | | | | | | | | | |
| **S1** | 1.90E-11 | 6.39E-02 | 1.00 | 7.09E-01 | 7.60E-05 | 1.26E-02 | 4.88E-01 | 9.20E-05 | 6.30E-05 | 1.14E-07 | 6.22E-10 | 7.19E-10 | 1.70E-04 | 1.95E-09 | 5.50E-04 | 1.06E-02 |
| **S2** | 2.16E-11 | 7.26E-02 | 1.14 | 8.05E-01 | 8.63E-05 | 1.43E-02 | 5.54E-01 | 1.05E-04 | 7.16E-05 | 1.30E-07 | 7.06E-10 | 8.17E-10 | 1.93E-04 | 2.21E-09 | 6.24E-04 | 1.21E-02 |
| **S3** | 1.74E-11 | 5.84E-02 | 0.91 | 6.48E-01 | 6.94E-05 | 1.15E-02 | 4.46E-01 | 8.41E-05 | 5.76E-05 | 1.04E-07 | 5.68E-10 | 6.57E-10 | 1.55E-04 | 1.78E-09 | 5.02E-04 | 9.70E-03 |
| **S4** | 2.49E-11 | 8.37E-02 | 1.31 | 9.29E-01 | 9.95E-05 | 1.65E-02 | 6.39E-01 | 1.21E-04 | 8.26E-05 | 1.49E-07 | 8.15E-10 | 9.42E-10 | 2.23E-04 | 2.55E-09 | 7.20E-04 | 1.39E-02 |
| **S5** | 1.61E-11 | 5.41E-02 | 0.85 | 6.00E-01 | 6.43E-05 | 1.06E-02 | 4.13E-01 | 7.79E-05 | 5.34E-05 | 9.66E-08 | 5.26E-10 | 6.09E-10 | 1.44E-04 | 1.65E-09 | 4.65E-04 | 8.99E-03 |
| **S6** | 1.61E-11 | 5.40E-02 | 0.85 | 5.99E-01 | 6.42E-05 | 1.06E-02 | 4.12E-01 | 7.78E-05 | 5.33E-05 | 9.65E-08 | 5.26E-10 | 6.08E-10 | 1.44E-04 | 1.65E-09 | 4.65E-04 | 8.97E-03 |

References

Borjan D, Knez Ž and Knez M (2021) Recycling of Carbon Fiber-Reinforced Composites-Difficulties and Future Perspectives. *Materials (Basel, Switzerland)* 14(15). DOI: 10.3390/ma14154191.

Goyal A and Nayak R (2020) Sustainability in yarn manufacturing. In: Nayak R (ed) *Sustainable technologies for fashion and textiles:* Duxford, Cambridge, Kidlington: Elsevier WP Woodhead Publishing, pp. 33–55.

Hohmann A (2019) Ökobilanzielle Untersuchung von Herstellungsverfahren für CFK-Strukturen zur Identifikation von Optimierungspotentialen, Technische Universität München.

Karuppannan Gopalraj S, Deviatkin I, Horttanainen M, et al. (2021) Life Cycle Assessment of a Thermal Recycling Process as an Alternative to Existing CFRP and GFRP Composite Wastes Management Options. *Polymers* 13(24): 4430. DOI: 10.3390/polym13244430.

López FA, Rodríguez O, Alguacil FJ, et al. (2013) Recovery of carbon fibres by the thermolysis and gasification of waste prepreg. *Journal of Analytical and Applied Pyrolysis* 104: 675–683. DOI: 10.1016/j.jaap.2013.04.012.

Mazzocchetti L, Benelli T, D’Angelo E, et al. (2018) Validation of carbon fibers recycling by pyro-gasification: The influence of oxidation conditions to obtain clean fibers and promote fiber/matrix adhesion in epoxy composites. *Composites Part A: Applied Science and Manufacturing* 112: 504–514. DOI: 10.1016/j.compositesa.2018.07.007.

Naqvi SR, Prabhakara HM, Bramer EA, et al. (2018) A critical review on recycling of end-of-life carbon fibre/glass fibre reinforced composites waste using pyrolysis towards a circular economy. *Resources, Conservation and Recycling* 136: 118–129. DOI: 10.1016/j.resconrec.2018.04.013.

Pimenta S and Pinho ST (2011) Recycling carbon fibre reinforced polymers for structural applications: technology review and market outlook. *Waste management (New York, N.Y.)* 31(2): 378–392. DOI: 10.1016/j.wasman.2010.09.019.

Vo Dong PA, Azzaro-Pantel C and Cadene A-L (2018) Economic and environmental assessment of recovery and disposal pathways for CFRP waste management. *Resources, Conservation and Recycling* 133: 63–75. DOI: 10.1016/j.resconrec.2018.01.024.

Wang Y, Li A, Zhang S, et al. (2023) A review on new methods of recycling waste carbon fiber and its application in construction and industry. *Construction and Building Materials* 367: 130301. DOI: 10.1016/j.conbuildmat.2023.130301.

Wei Y and Hadigheh SA (2022) Cost benefit and life cycle analysis of CFRP and GFRP waste treatment methods. *Construction and Building Materials* 348: 128654. DOI: 10.1016/j.conbuildmat.2022.128654.
